# Two-Stage Unet with Gated-Conv Fusion for Binaural Audio Synthesis

**DOI:** 10.3390/s25061790

**Published:** 2025-03-13

**Authors:** Wenjie Zhang, Changjun He, Yinghan Cao, Shiyun Xu, Mingjiang Wang

**Affiliations:** Key Laboratory for Key Technologies of IoT Terminals, Harbin Institute of Technology (Shenzhen), Shenzhen 518055, China; 23s152109@stu.hit.edu.cn (W.Z.);

**Keywords:** binaural audio synthesis, spatial perception, self-attention, UNet

## Abstract

Binaural audio is crucial for creating immersive auditory experiences. However, due to the high cost and technical complexity of capturing binaural audio in real-world environments, there has been increasing interest in synthesizing binaural audio from monaural sources. In this paper, we propose a two-stage framework for binaural audio synthesis. Specifically, monaural audio is initially transformed into a preliminary binaural signal, and the shared common portion across the left and right channels, as well as the distinct differential portion in each channel, are extracted. Subsequently, the POS-ORI self-attention module (POSA) is introduced to integrate spatial information of the sound sources and capture their motion. Based on this representation, the common and differential components are separately reconstructed. The gated-convolutional fusion module (GCFM) is then employed to combine the reconstructed components and generate the final binaural audio. Experimental results demonstrate that the proposed method can accurately synthesize binaural audio and achieves state-of-the-art performance in phase estimation (Phase-$l_{2}$: 0.789, Wave-$l_{2}$: 0.147, Amplitude-$l_{2}$: 0.036).

## 1. Introduction

With the rapid development of applications such as virtual reality (VR), augmented reality (AR), and gaming, there has been an increasing emphasis on immersive experiences. Spatial audio, as a medium that enhances the sense of presence, has attracted significant attention [1]. In real-world scenarios, recording spatial audio is often difficult due to the substantial resources and labor costs required, whereas monaural audio is much easier to obtain and involves minimal resource consumption. However, monaural audio, due to its limited input dimensions, captures the audio only at a single spatial point, preventing listeners from perceiving spatial positions. Based on this understanding, directly rendering binaural spatial audio from monaural audio is essential. This approach not only reduces computational costs but also enables independent audio streams for each audio object with definable spatial positions, enabling listeners to experience immersive environments in applications such as AR/VR [2,3].

**Figure 1:**
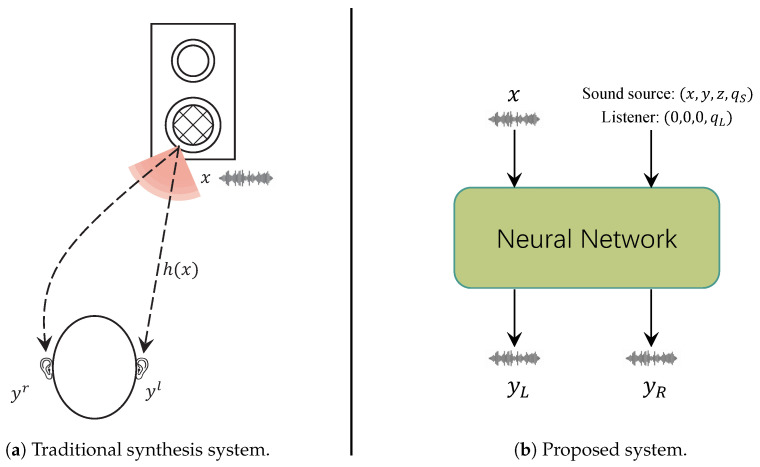
Binaural synthesis system.

Considering the process by which sound travels to a listener’s ears, sound undergoes diffuse reflections within the room and interacts with the listener’s pinnae, head, torso, and the surrounding sound field. These interactions create differences in the time (Interaural Time Difference, ITD) and level (Interaural Level Difference, ILD) at which sound reaches each ear. These auditory localization cues help listeners determine the position of the sound source [4,5], providing the impression that the sound source is located within a 3D space (see Figure 1a).

In the task of rendering monaural audio into binaural audio, the primary objective is to reconstruct these auditory localization cues. Traditional methods primarily rely on digital signal processing (DSP) techniques to model spatial cues through head-related impulse responses (HRIRs), binaural room impulse responses (BRIRs), environmental noise, or reverberation to render binaural audio [6,7]. Sunder et al. combined head-related transfer functions (HRTFs) with head tracking and room acoustics modeling to render binaural audio [6]. Zotkin et al. employed HRTF interpolation and techniques for reproducing room impulse responses to achieve audio rendering [8]. More recently, Zamir et al. applied phase correction to improve individual head-related transfer functions for binaural audio reproduction [9]. However, due to the nonlinear nature of sound propagation and the inability of DSP systems to accurately model all corresponding interactions, these methods are often insufficient to faithfully reproduce real-world audio [10,11].

While highlighting these limitations, recent studies have introduced neural networks into the field of audio processing. With their strong capability to model non-linear relationships, deep learning networks have been widely applied in areas such as speech enhancement [12,13,14,15,16] and synthesis. Given their success in audio-related tasks, numerous neural network-based binaural audio synthesis methods have been proposed [10,17,18,19,20,21]. For instance, [22] first introduced a framework that combines video frames to reconstruct binaural audio. Zhou et al. [23] integrated separation tasks with audio spatialization. Similarly, Xu et al. [24] utilized spherical harmonics decomposition and head-related impulse responses to capture the relationship between spatial positions and the corresponding received binaural audio. However, these methods, being video-frame-driven, often fail to generalize well across all scenarios, such as cases where audio is presented without accompanying video. Based on these insights, end-to-end rendering of binaural audio has garnered increasing attention. Methods such as [10,11,25,26] have achieved rendering from monaural audio to binaural audio. However, it should be noted that these approaches face limitations when estimating the phase of binaural audio, resulting in suboptimal performance when reconstructing spatial localization cues such as ITD and ILD [27].

**Figure 2:**
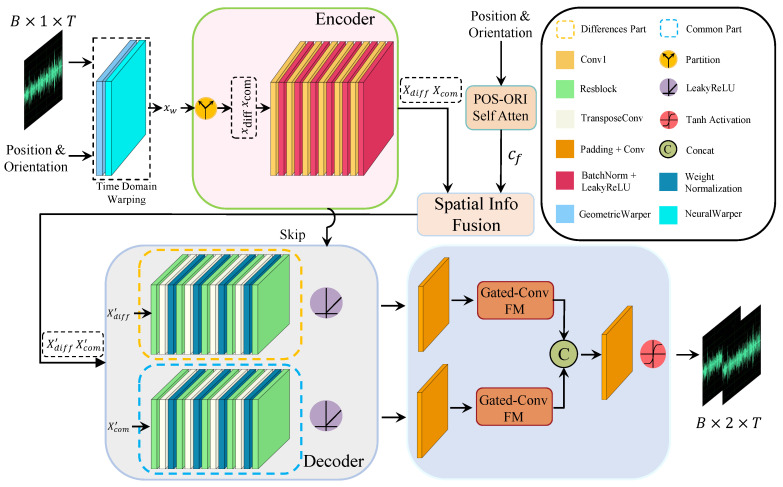
Overall architecture of proposed system for binaural audio synthesis.

In this work, we propose a two-stage U-Net with gated-conv fusion for binaural audio synthesis, focusing primarily on source motion and interaural differences. Unlike previous approaches, we provide a novel perspective on the problem. The synthesis of binaural audio consists of two main processes: (1) Monaural audio is emitted by the sound source, and if it were to propagate without any modifications, the audio arriving at the left and right ears would be identical. (2) The similar audio is then altered by BRIRs and HRTFs, which introduce differences in the perceived audio between the left and right ears [4,28]. Therefore, we posit that binaural audio can be divided into common and differential portions. Based on this representation, we propose a novel framework to process these distinct aspects, as illustrated in Figure 2. Our main contributions are summarized as follows:

- We decompose the binaural audio representation into two components to accurately model the sound propagation process, which are separately extracted and reconstructed based on time-domain warping.
- To enhance the accuracy of the synthesized binaural audio, we propose the gated-conv fusion module (GCFM) to integrate the reconstructed components by suppressing less informative features, allowing only the useful information to be utilized for further binaural audio synthesis.
- To capture the spatial perception induced by source movement, we employ a position–orientation self-attention module (POS-ORI self-attention, POSA), which effectively combines audio features with spatial localization cues.

The structure of this paper is organized as follows: Section 2 reviews the related work relevant to this study. The signal model for binaural audio synthesis, the overall framework of the proposed model, and detailed descriptions of its submodules are presented in Section 3. Section 4 introduces the datasets used in the experiments, describes the experimental setup, and outlines the objective and subjective metrics employed for performance evaluation. In Section 5, we analyze the performance of the proposed model and compare it with other existing models. Finally, the conclusions are provided in Section 6.

## 2. Related Work

### 2.1. Binaural Audio Synthesis

Traditional binaural audio synthesis methods restore the sound propagation process through digital signal processing. In [29], binaural audio is synthesized by modeling the distortion effects caused by the interaction of sound with physical objects during propagation. Sunder et al. combine HRTFs with head tracking and room acoustic modeling to render binaural audio [6]. However, traditional methods often build generalized functions, which may not meet the specific requirements of specialized scenarios.

Several approaches using neural networks for binaural audio synthesis have been proposed. Gebru et al. [18] utilized neural networks to learn HRTFs, while Gao et al. [17] combined video frame information to restore the position of the sound source for binaural audio synthesis. In this work, we use monaural audio without relying on video frames to synthesize binaural audio.

### 2.2. UNet

UNet was initially proposed by Ronneberger et al. [30], primarily for biomedical image segmentation tasks. Due to its flexible structure and superior performance, UNet has been widely applied across various domains that require precise localization and context preservation. UNet adopts an encoder–decoder architecture and facilitates efficient feature transfer through skip connections. Its design objective is to simultaneously capture high-level semantic information and spatial details. Given its outstanding performance, UNet has also been applied to audio processing tasks. In [31], an auxiliary encoder was used to extract relevant features of distant speech, and an attention mechanism was employed to suppress echo in mixed speech by selectively extracting features. An end-to-end speech enhancement method was designed based on Wave-unet in [32]. Nair et al. [33] proposed a cascaded structure combining time-domain T-UNet and time–frequency-domain TF-UNet to address various distortion issues in speech enhancement. To the best of our knowledge, UNet has only been used for binaural audio synthesis tasks when guided by visual frames [20,22,23].

### 2.3. Attention Mechanism

The attention mechanism allocates different weights based on the importance of different parts of the input, thereby enhancing the model’s efficiency and accuracy [34,35,36]. As noted in [37], it is the first to handle input and output sequences of varying lengths, providing a foundation for training the correspondence between inputs and outputs, which underpins the attention mechanism. Cross-attention, as introduced in [38], effectively fuses different types of data by using queries from one sequence or modality, while keys and values are derived from another sequence or modality.

## 3. Method

### 3.1. Signal Model

In real-life scenarios, factors such as room reverberation and head shadowing effects lead to perceptual differences between the left and right ears. As illustrated in Figure 1a, the sound emitted by the source is denoted as *x*, while the sounds received by the left and right ears are denoted as $y^{l}$ and $y^{r}$, respectively. The system response function of the entire propagation path is represented as *h*, and the relationship can be expressed as follows:

(1) $$\left(y^{l},y^{r}\right)=h\left(x\right)$$

However, due to differences in spatial position, listeners in other positions cannot experience the same perception. We aim to synthesize binaural audio from monaural audio using a neural network approach to achieve a sense of presence, which is shown in Figure 1b. Based on this, we seek to construct a model *f* to reconstruct the sound propagation process *h*, which can be expressed as follows:

(2) $$\left(y^{l},y^{r}\right)=f\left(x,\;c\right)$$

Here, *c* represents the spatial relationship between the sound source and the listener, encompassing positional coordinates (x,y,z). Additionally, $q_{s}$ and $q_{l}$ are expressed as quaternions, representing the orientation of the sound source and the listener, respectively, thereby providing a comprehensive and singularity-free representation of their angular alignment in space. Based on the above analysis, the task of this paper is to synthesize binaural audio $\left(y^{l},y^{r}\right)$ from monaural audio *x* using the spatial positioning information between the sound source and the listener.

### 3.2. Overall Structure

In recent years, UNet has shown excellent performance in binaural audio synthesis tasks guided by visual frames. On this basis, in order to improve the accuracy of binaural audio synthesis, we use a modified UNet to render the monaural audio $x\in R^{B\times 1\times T}$, where *B* represents the batch size and *T* is the length of the audio sequence, into binaural audio $y=\left(y^{l},y^{r}\right)\in R^{B\times 2\times T}$. The completed structure of this method is shown in Figure 2.

Our method is divided into two stages to synthesize binaural audio and enhance the spatial perception of binaural audio. In the first stage, we follow the time warping module (TW) in [10], where mono audio *x* is initially warped through TW. The spatial position and azimuth information of the sound source $c\in R^{B\times 7\times L}$, where 7 includes position information from three channels and azimuth information from four channels representing the direction of the listener’s head and expressed in quaternions [39] and *L* represents the number of different positions that the sound source occupies within the time range *T*. This information is used as a conditioning input to the TW module. The module synthesizes the warped binaural audio $x_{w}=\left(x_{w}^{l},x_{w}^{r}\right)\in R^{B\times 2\times T}$, using a nonparametric geometric distortion field and a trainable neural distortion field. It is important to note that due to the clear monotonic causal relationship between the mono signal emitted by the sound source and the signal received by the ears, the distortion field both maintains this causal relationship and preserves monotonicity.

The ITD and ILD play a crucial role in our ability to localize sound sources. ITD arises due to the spatial disparity between the two ears relative to the sound source, causing a temporal delay in the arrival of the sound signal at each ear. On the other hand, ILD is induced by the head shadowing effects. Based on this, we can consider the differential portion (DP) between the sound signals received by the two ears as the key factor for determining the sound source’s direction, while the common portion (CP) represents the similar part of the sound signals received by both ears. This process can be expressed as follows:

(3) $$x_{\mathrm{com}}=\frac{x_{w}^{l}+x_{w}^{r}}{2},\;x_{\mathrm{diff}}=\frac{x_{w}^{l}-x_{w}^{r}}{2}$$

where $x_{\mathrm{com}}$ and $x_{\mathrm{diff}}$ represent the CP and DP, respectively. $x^{l}$ and $x^{r}$ denote the signals of the left and right channels after warping, respectively.

In the second stage, unlike traditional binaural audio synthesis encoding methods, the initially distorted binaural audio is decoupled into $x_{\mathrm{diff}}$ and $x_{\mathrm{com}}$, which are then fed into a subsequent UNet for further feature extraction and reconstruction. During the downsampling phase, both $x_{\mathrm{diff}}$ and $x_{\mathrm{com}}$ undergo the same downsampling operation to generate $X_{\mathrm{diff}}$ and $X_{\mathrm{com}}$, respectively. These are then fused and concatenated with spatial position information processed by POSA, resulting in $X_{\mathrm{diff}}^{\prime }$ and $X_{\mathrm{com}}^{\prime }$. On this basis, $X_{\mathrm{diff}}^{\prime }$ and $X_{\mathrm{com}}^{\prime }$ are each subjected to multiple upsampling operations with residual connections, facilitating the decoder’s reconstruction of both components. To allow useful information to propagate further, the upsampled results are passed through GCFM, and the two pieces of information are concatenated. Finally, the output is passed through a conv1 layer followed by a tanh activation function to produce the final binaural audio output $y=(y^{l},y^{r})$.

### 3.3. Downsampling and Upsampling

**Figure 3:**
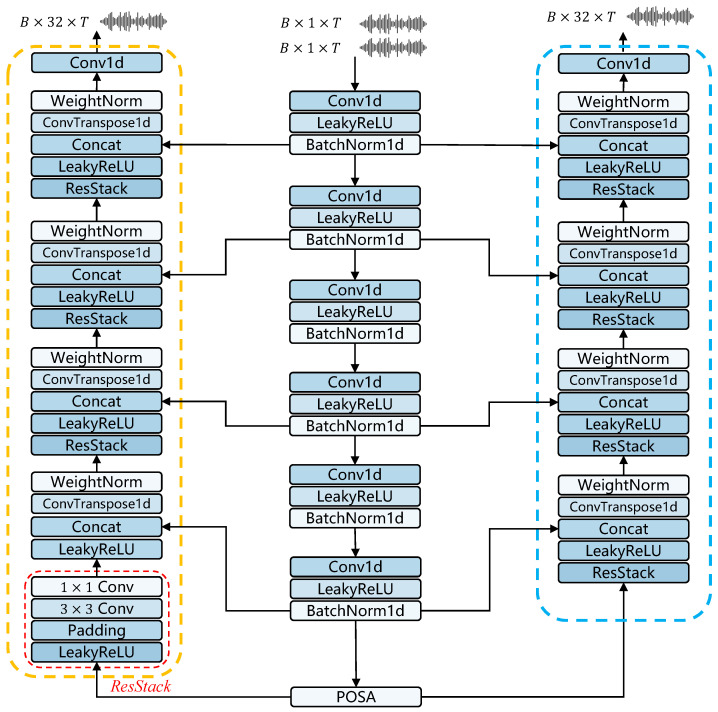
Overall architecture of asymmetric UNet structure.

In the extraction and reconstruction of binaural audio features, we adopt a UNet-style architecture. However, unlike the basic UNet structure, the proposed architecture incorporates asymmetric downsampling and upsampling operations. Experimental results indicate that fewer upsampling steps not only save computational resources but also prevent the loss of spatial information. Due to the fact that we separately reconstruct $x_{\mathrm{com}}$ and $x_{\mathrm{diff}}$, and by reducing the number of upsampling steps, we minimize over-reconstruction, thus preserving more crucial spatial information. Building on these insights, the feature extraction phase of the audio consists of six downsampling operations, while the reconstruction phase includes only four upsampling operations. Specifically, we use transposed convolution for upsampling, as it allows the model to automatically learn how to effectively expand spatial dimensions according to the data. However, it is important to note that inappropriate convolution kernels can lead to ’checkerboard’ patterns, as noted in [40]. To avoid this, we meticulously design the kernels, strides, and padding for the transposed convolution operations. Furthermore, after each upsampling operation, a ResStack module is introduced to integrate audio features and enhance local correlations. Subsequently, the reconstructed features are concatenated along the channel dimension with features from skip connections after passing through an activation function. The asymmetric UNet structure is illustrated in Figure 3.

### 3.4. POS-ORI Self-Attention Module

Previous studies have shown that in binaural audio synthesis tasks, particular attention is often paid to whether the synthesized binaural audio exhibits spatial awareness [17,18]. As such, the position and orientation between the sound source in space and the listeners become crucial. In the proposed method, we introduce POSA to effectively extract the spatial features of *c* in Equation (2). Once the spatial features are extracted, they are combined with the audio features obtained through downsampling, enabling the audio to focus on moments where the position changes.

**Figure 4:**
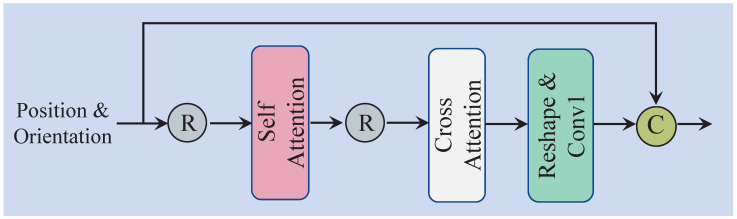
Overall architecture of POS-ORI self-attention module.

The overall structure of POSA is depicted in Figure 4. The input to the module consists of spatial information, specifically position and orientation, represented as $c\in R^{B\times 7\times L}$. Initially, the spatial information is reshaped and passed through the self-attention module to generate $c_{s}$. The self-attention mechanism (SA) reallocates attention weights, allowing the model to focus on the critical moments when the sound source undergoes significant changes. Moreover, since both the relative position and orientation between the sound source and the listener in space vary simultaneously, we incorporate cross-attention (CA) to enable the model to effectively integrate both types of information, resulting in $c_{c}$. Subsequently, a reshape operation and a Conv1d layer are applied. Finally, through a residual connection, $c_{c}$ and *c* are integrated to produce the final output $c_{f}$. This process can be formalized as follows:

(4) $$\begin{array}{cc} c_{f} & =\mathrm{Concat}(c_{c},\;c)\\ \; & =\mathrm{Concat}(\mathrm{CA}\left(c_{s}\right),\;c)\\ \; & =\mathrm{Concat}(\mathrm{CA}(\mathrm{SA}(\mathrm{Reshape}(c))),\;c) \end{array}$$

**Figure 5:**
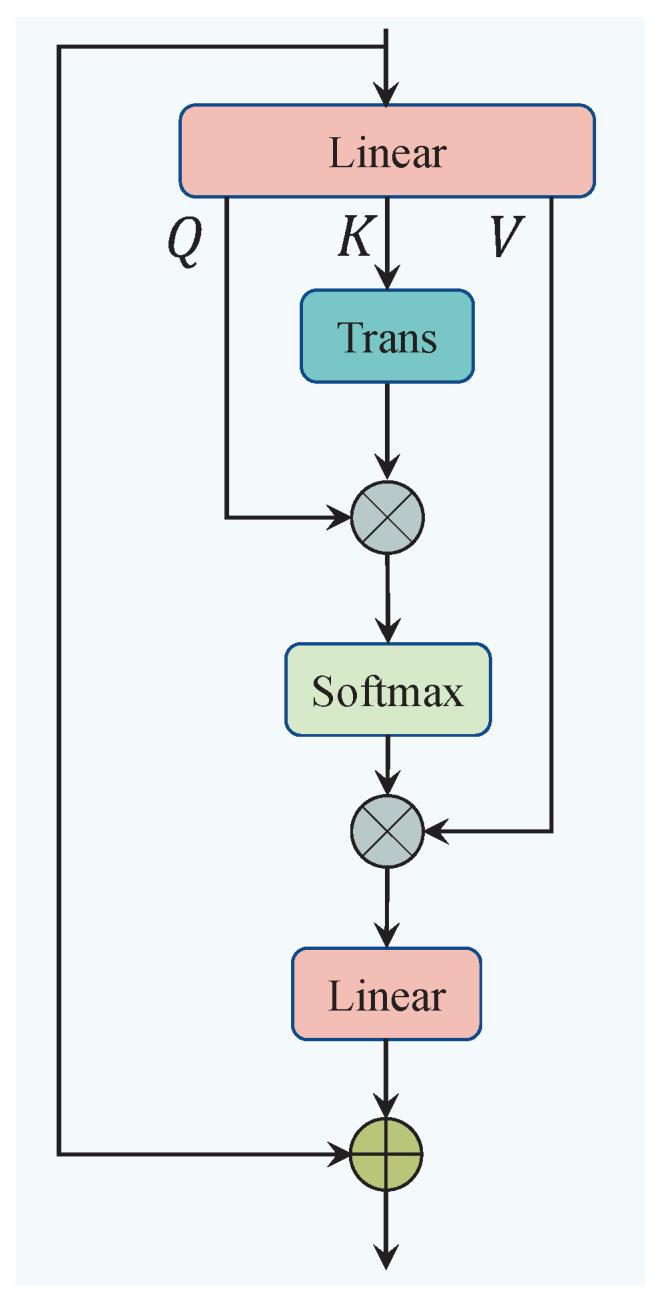
The architecture of the self-attention module (SA).

In terms of the individual components, SA helps the network capture long-range dependencies within the internal features. The process of SA is illustrated in Figure 5. Specifically, SA first reshapes the spatial information into $c_{r}\in R^{B\times L\times 7}$, and then applies three linear mappings to generate the query ($Q\in R^{B\times L\times D}$), key ($K\in R^{B\times L\times D}$), and value ($V\in R^{B\times L\times D}$), where *D* represents the encoded dimension. Second, the query and key are multiplied to compute the attention weights, which are used to select important parts of the spatial information. The attention weights are then normalized using a Softmax function, and the result is multiplied by the value *V*. This entire process is expressed as follows:

(5) $$\mathrm{SA}\left(Q,K,V\right)=\mathrm{Softmax}\left(\frac{Q\times K^{T}}{\sqrt{D}}\right)\times V$$

Third, an output linear layer is employed to map the attention result back to seven dimensions, with a residual connection applied to stabilize the output of the self-attention mechanism. The complete process is as follows, see Figure 5:

(6) $$\begin{array}{cc} c_{s} & =\mathrm{Linear}(\mathrm{SA}(Q,K,V))+\mathrm{Reshape}(c)\\ \; & =\mathrm{Linear}(\mathrm{SA}(\mathrm{Linear}(c),\mathrm{Linear}(c),\mathrm{Linear}(c)))+\mathrm{Reshape}(c) \end{array}$$

**Figure 6:**
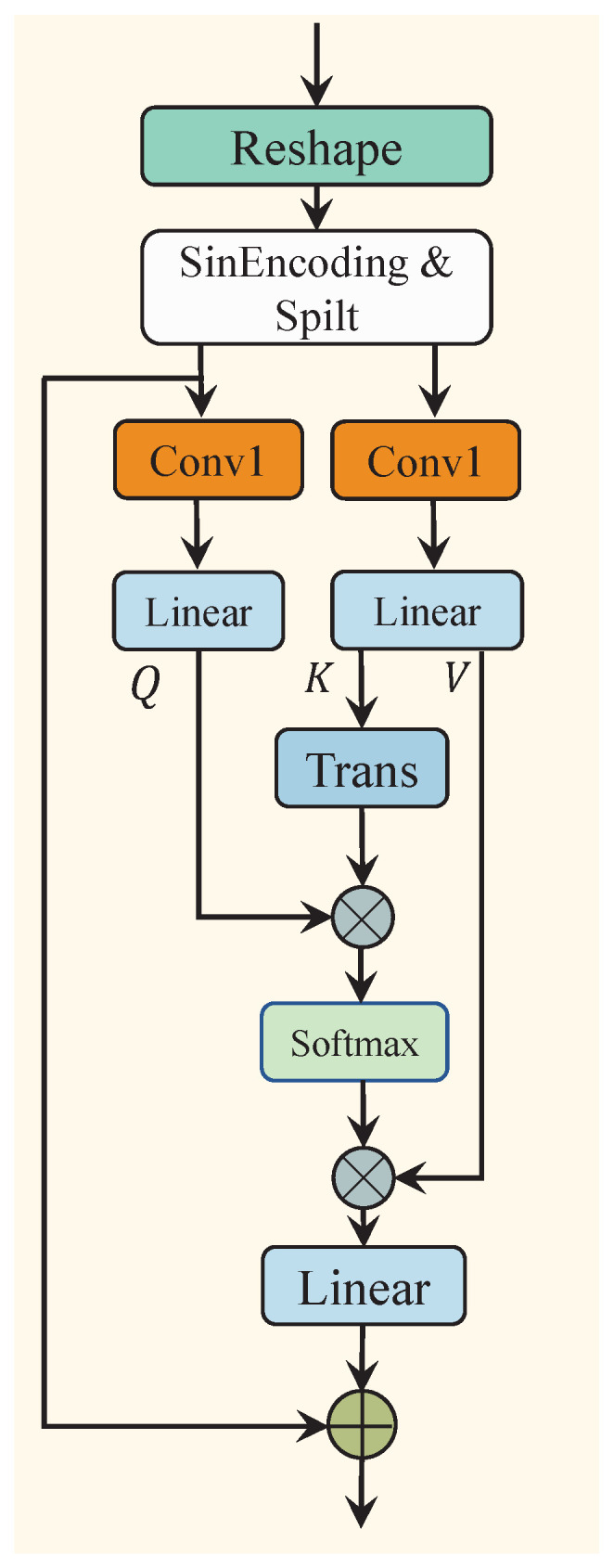
The architecture of the cross-attention module (CA).

Furthermore, since the relative position (Pos) and orientation (Ori) information between the sound source and the listener change simultaneously, we employ Cross-Attention (CA) to capture the correlation between Pos and Ori. First, the output of the self-attention mechanism, $c_{s}$, is reshaped and sinusoidal-encoded to maintain the original sequence order. The sinusoidal-encoded result is then split into position encoding information $E_{POS}$ and orientation encoding information $E_{ORI}$. Next, a Conv1d layer is applied to unify the channel dimensions of both types of information to $D_{E}$. In the subsequent step, the position encoding information is mapped to query ($Q\in R^{\left(BL\right)\times D_{E}\times D}$) through a linear layer, while the angle encoding information is mapped to key ($K\in R^{\left(BL\right)\times D_{E}\times D}$) and value ($V\in R^{\left(BL\right)\times D_{E}\times D}$). The output is then computed through the process described in Equation (5). Finally, through a residual connection, $E_{POS}$ is integrated with the output to obtain $c_{c}$. The entire process is expressed as follows, see Figure 6:

(7) $$c_{c}=\mathrm{Linear}\left(\mathrm{SA}\left(Q,K,V\right)\right)+E_{\mathrm{POS}}$$

### 3.5. Spatial Information Fusion Module

To enhance the spatial perception and accuracy of the synthesized binaural audio, and to guide the encoded audio features based on the position and orientation of the sound source, we introduce a spatial information fusion module. To ensure that the feature dimensions of the results obtained from the POSA align with the downsampled audio features, we apply a linear interpolation operation. Subsequently, the two feature sets are combined and passed through a Conv1d layer to produce the final outputs $\left(X_{\mathrm{diff}}^{\prime },\;X_{\mathrm{com}}^{\prime }\right)$. The entire process is described as follows:

(8) $$\begin{array}{cc} \; & X_{\mathrm{diff}}^{\prime }=\mathrm{Conv}1\left(\mathrm{Concat}\left(X_{\mathrm{diff}},\;\mathrm{Inter}\left(\mathrm{Conv}1\left(c_{f}\right)\right)\right)\right) \end{array}$$

(9) $$\begin{array}{cc} \; & X_{\mathrm{com}}^{\prime }=\mathrm{Conv}1\left(\mathrm{Concat}\left(X_{\mathrm{com}},\;\mathrm{Inter}\left(\mathrm{Conv}1\left(c_{f}\right)\right)\right)\right) \end{array}$$

**Figure 7:**
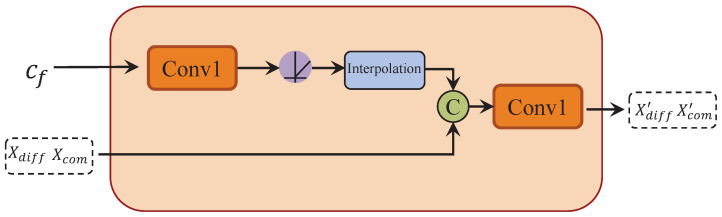
Structure diagram of spatial information fusion module.

Here, $X_{\mathrm{diff}}$ and $X_{\mathrm{com}}$ refer to the downsampled audio features, while Inter represents the interpolation operation for feature dimension alignment. Conv1 represents a 1D convolution operation. See Figure 7.

### 3.6. Gated-Conv Fusion Module

**Figure 8:**
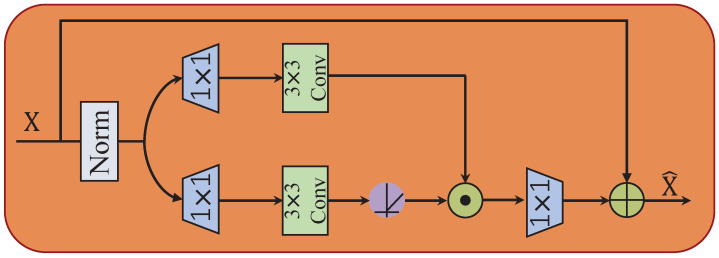
Structure diagram of Gated-Conv fusion module.

In previous work [19,20], audio information is directly obtained by the upsampling operation. In this study, we introduce a gating mechanism after the upsampling process to further extract key information from the audio features obtained by the upsampling operation. The architecture of our GCFM is illustrated in Figure 8. The gating mechanism (Gating) is formulated as the element-wise product of two parallel paths of linear transformation layers. Given an input tensor *X*, GCFM can be expressed as follows:

(10) $$\begin{array}{cc} \mathrm{GCFM}(X) & =\mathrm{Gating}(X)+X\\ \; & =Conv_{1}\left(\psi \left(Conv_{3}\left(Conv_{1}\left(\phi \left(X\right)\right)\right)\right)⊙Conv_{3}\left(Concv_{1}\left(\phi \left(X\right)\right)\right)\right)+X \end{array}$$

where ⊙ denotes element-wise multiplication. ψ refers to the non-linear activation function, $Conv_{\left(\mu \right)}$ represents an μ×μ convolution, and ϕ represents the normalization operation. In general, GCFM controls the flow of information through the gated unit after upsampling, allowing it to filter out important features. That is, GCFM performs a distinct role compared to conventional upsampling. Since GCFM performs more operations and processes more information after upsampling, we select the encoding dimension in GCFM to be the same as the input dimension to reduce the compute burden.

### 3.7. Loss Function

In binaural audio synthesis tasks, the importance of both magnitude and phase information cannot be overlooked. Previous studies have shown a strong correlation between phase errors and the noise and distortion present in synthesized binaural audio [41]. Therefore, accurate phase estimation is crucial for guiding the synthesis of binaural audio. On the other hand, after optimizing the waveform using a temporal $l_{2}$ loss, the magnitude fitting performs well. Based on these insights, we choose to optimize the proposed model by minimizing a weighted sum of the $l_{2}$ loss and phase loss between the synthesized binaural audio *y* and the ground truth $y_{t}$. The loss function we use is defined as follows:

(11) $$L=\lambda_{1}\cdot L_{2}\left(y,y_{t}\right)+\lambda_{2}\cdot L_{\mathrm{phase}}\left(y,y_{t}\right)$$

where $\lambda_{1}$ and $\lambda_{2}$ represent the weights for the $l_{2}$ loss and phase loss, respectively. $L_{2}\left(y,y_{t}\right)$ represents the mean squared error (MSE) between the synthesized binaural audio *y* and the ground truth audio $y_{t}$, while $L_{\mathrm{phase}}\left(y,y_{t}\right)$ represents the phase error between the synthesized and the ground truth audio. The two error functions are presented as follows:

(12) $$\begin{array}{cc} \; & L_{2}\left(y,y_{t}\right)=\frac{1}{n}\sum^{n}{\left(y-y_{t}\right)}^{2} \end{array}$$

(13) $$\begin{array}{cc} \; & L_{\mathrm{phase}}\left(y,y_{t}\right)=L\left(P_{\mathrm{STFT}(y)},P_{\mathrm{STFT}(y_{t})}\right) \end{array}$$

Here, *n* represents the length of the audio sequence, STFT denotes the Short-Time Fourier Transform operation, and *P* represents the phase calculation.

## 4. Experiment

### 4.1. Datasets

In our experiments, we utilize a binaural speech dataset published in the literature [10] for training and testing. The dataset includes recordings from 8 different speakers, consisting of 4 males and 4 females. The listener is represented by a human body model equipped with binaural microphones. The subjects are instructed to move around the KEMAR mannequin and speak within a radius of 1.5 m. A total of 2 h of paired monaural and binaural data at 48 kHz were recorded, while the positions and orientations of the speaker and KEMAR mannequin were sampled at a rate of 120 Hz. Additionally, the OptiTrack system was used to capture the position and orientation of both the sound source and the mannequin. Each position and orientation are represented by 7 channels, with 3 channels for position and 4 channels for orientation. This is currently the only publicly available dataset recorded outside of an anechoic chamber. For consistency and ease of comparison, we follow the original training, validation, and test set splits described in [26].

### 4.2. Implementation Details

In our experiments, we selected a batch size of 32, with each batch consisting of 200 ms of audio during training. For the downsampling phase, the kernel size, stride, and padding were set to 4, 2, and 1, respectively. For the first and second upsampling layers, the kernel size, stride, and padding were set to 8, 4, and 2, respectively. For the remaining upsampling layers, the kernel size, stride, and padding were set to 4, 2, and 1, respectively. The changes in the shape of the audio features during upsampling and downsampling operations are presented in Table 1.

In the POSA module, the first Conv1 layer is configured with 10 input channels and 64 output channels, utilizing a kernel size of 3 and a padding of 1. The second Conv1 layer is designed with 576 input channels and 256 output channels, employing a kernel size of 7, while all other parameters remain at their default settings. The specific shape changes are shown in Table 2. Before the GCFM, the input and output channels of the 1D conv are set to 32 and 8, respectively. After applying the GCFM, the input and output channels of the 1D conv are set to 16 and 2, respectively.

The optimizer used in the experiment is Adam [42], with a maximum learning rate of $1\times 10^{-3}$ and a minimum learning rate of $1\times 10^{-6}$. The learning rate is adjusted by the Cosine Annealing strategy [43]. A linear warmup with a step size of 1000 is applied. The weight parameters $\lambda_{1}$ and $\lambda_{2}$ are set to 1.0 and 0.01 in the loss function, respectively. The model is trained for a total of 100 epochs.

### 4.3. Evaluation Metrics

In terms of improving the listener’s experience, we conducted both quantitative and perceptual evaluations of the binaural audio synthesized by the model. Our approach involved training and evaluation based on the following quantitative metrics:

- Wave-$l_{2}$: The MSE in the temporal waveform between the synthesized binaural audio and the ground truth.
- Phase-$l_{2}$: The MSE in the phase component between the synthesized binaural audio and the ground truth after applying STFT, which provides an indication of the accuracy of the synthesized audio’s ITD.
- Amplitude-$l_{2}$: The MSE in the amplitude between the synthesized binaural audio and the ground truth after STFT, which serves as a measure of the accuracy of ILD in the synthesized audio.

In the case of STFT, the FFT size is set to 4096, the window length is configured to 1920, the hop length is set to 480, and the windowing function employed is the Hamming window. In addition to the aforementioned metrics, the mean opinion scores (MOS) provided by the participants, representing various perceptual aspects, are also considered in the evaluation to assess the audio quality intuitively.

- MOS: Overall naturalness and clarity of the audio.
- Spatialization MOS: The spatial perception of the audio.
- Similarity MOS: The similarity between the synthesized binaural audio and the ground truth.

## 5. Results and Analysis

### 5.1. Performance Comparison of Binaural Audio Synthesis Models

#### 5.1.1. Quantitative Evaluation

We report a comparison of the quantitative evaluation metrics of our method and other binaural audio synthesis models in Table 3. Except for NFS, we adjust their hyper-parameters to make the number of parameters comparable with our model to ensure fair comparisons. In addition to this, the quantitative evaluation metrics of the traditional DSP method introduced in [10] are also presented. We observe that our proposed method outperforms other models in phase estimation, indicating that our approach synthesizes binaural waveforms that are closer to the real audio. Additionally, it achieves the second-best scores in the Wave-$l_{2}$ metrics. In summary, the objective metrics demonstrate that the proposed method shows superior performance compared to the classic WarpNet baseline.

**Figure 9:**
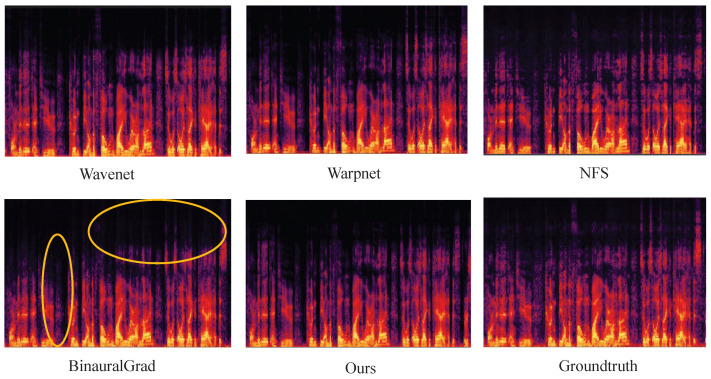
The spectrograms of binaural audio synthesized by each model. (We present the spectrogram for the left ear as an example).

In addition to testing on the binaural audio test set, we also evaluated the binauralization of out-of-domain audio. Figure 9 presents the spectrograms of binaural audio synthesized by different models, along with the spectrogram of the ground truth audio. In Figure 9, the spectrograms of the left ear are used as examples for comparison.

From Figure 9, we observe that although BinauralGrad demonstrates strong performance in terms of objective metrics, it exhibits significant spectral loss when compared to Groundtruth. This phenomenon can lead to a noticeable degradation in perceived audio quality at certain moments. In contrast, the higher accuracy achieved by our method ensures better preservation of audio quality during binaural audio synthesis. In addition, we uploaded the synthesized binaural audio online https://ethuil.github.io/Two_stage_Unet_with_Gated_conv_Fusion_for_Binaural_Audio_Synthesis (accessed on 9 March 2025).

In addition to the quantitative evaluation of the synthesized audio, it is essential to consider the computational cost of the model. Our approach demonstrates clear advantages over WarpNet and WaveNet, both in terms of quantitative performance and parameter efficiency. Although NFS has a smaller parameter count, its performance in the quantitative evaluation is notably inferior. While BinauralGrad exhibits strong performance in evaluation metrics, our method shows a significant advantage when accounting for computational cost, particularly in terms of multiply–accumulates (MACs). Specifically, our model has a comparable parameter count to WarpNet but only requires 14.3% of WarpNet’s computational complexity. The parameters and computational complexities for all models are summarized in Table 3.

#### 5.1.2. Perceptual Evaluation

Building on quantitative evaluation, we further conducted a perceptual evaluation of the synthesized binaural audio. In this experiment, 30 participants were invited to rate the synthesized binaural audio samples. Each participant evaluated five speech samples from the binaural speech dataset as well as five out-of-domain audio samples, including sounds such as footsteps, music, and songs. Participants were instructed to rate the samples based on overall naturalness, spatialization, and similarity. It is worth noting that the similarity metric is not required for out-of-domain audio samples.

The evaluation scores ranged from 1 to 5, with higher scores indicating better performance. The results of perceptual evaluation with a 95% confidence interval are presented in Table 4. As shown in the table, our method outperforms other models across all metrics, achieving the best overall performance.

**Figure 10:**
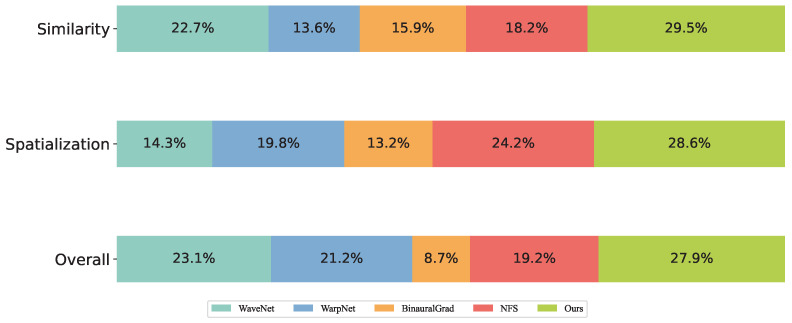
The proportion of highest scores awarded by participants across different models.

Furthermore, the proportions of audio samples receiving the highest scores for each model are presented in Figure 10. It can be observed that the proposed method achieves the highest proportions across all evaluated aspects. In other words, the binaural audio synthesized by the proposed model demonstrates superior performance across all dimensions compared to other models.

### 5.2. Ablation Study

We conducted ablation studies to investigate the role of each module in the proposed model by selectively removing specific components. The results of the ablation experiments are presented in Table 5. only TW indicates the use of only the TW module for distorting monaural audio without employing deeper levels of encoding and decoding. The results clearly show that the binaural audio synthesized using only the TW module performs poorly across all metrics. w/o POSA signifies the removal of POSA from the bottleneck in the UNet framework. The results reveal that the loss of spatial information leads to an increase in Wave-$l_{2}$ and Phase-$l_{2}$, indicating that the synthesized binaural audio becomes less coherent. w/o GCFM denotes the replacement of the GCFM module in the final upsampling stage, where the upsampled information is directly combined to reconstruct binaural audio. The results demonstrate that merely combining upsampling information is insufficient to fully reconstruct the audio, resulting in degraded Wave-$l_{2}$ and Phase-$l_{2}$ metrics. These findings highlight that the inclusion of the GCFM module enables the model to effectively reconstruct the audio, significantly improving the spatialization quality of the synthesized binaural audio.

## 6. Conclusions

We propose a method for synthesizing monaural audio into binaural audio to provide a more immersive auditory experience. Considering the presence of ITD and ILD, we decompose the audio features into CP and DP, which are reconstructed separately. Furthermore, we introduce the GCFM module to integrate the reconstructed DP and CP components for generating binaural audio. Experimental results demonstrate that the proposed method achieves state-of-the-art performance in audio spatialization. As future work, we plan to explore strategies for improving the generalization capability of the model and enhancing the accuracy of high-frequency audio reconstruction.

## Figures and Tables

**Table 1:** The shapes of audio features during the downsampling and upsampling processes. (For brevity, the downsampling operation is denoted as DS, and the upsampling operation as US).

Layer	Input Size	Output Size
DS0	B×1×T	B×64×(T/2)
DS1	B×64×(T/2)	B×128×(T/4)
DS2	B×128×(T/4)	B×256×(T/8)
DS3	B×256×(T/8)	B×512×(T/16)
DS4	B×512×(T/16)	B×512×(T/32)
DS5	B×512×(T/32)	B×1024×(T/64)
US0	B×512×(T/64)	B×256×(T/16)
US1	B×256×(T/16)	B×128×(T/4)
US2	B×128×(T/4)	B×64×(T/2)
US3	B×64×(T/2)	B×32×T

**Table 2:** The shapes of audio features with the spatial information fusion module.

Layer	Input Size	Output Size
Conv1	B×10×L	B×64×L
Inter	B×64×L	B×64×(T/64)
Concat	B×64×(T/64)	B×(1024+64)×(T/64)
Conv1(after connect)	B×(1024+64)×(T/64)	B×512×(T/64)

**Table 3:** Comparison of quantitative evaluation metrics between our method and other binaural audio synthesis models.

Model	Year	Wave-$l_{2}$ ↓	Phase-$l_{2}$ ↓	Amplitude-$l_{2}$ ↓	#Param↓	MACs↓
DSP	-	0.485	1.388	0.058	-	-
WaveNet [25]	2016	0.179	0.968	0.037	4.65 M	22.34 G
WarpNet [10]	2021	0.167	0.807	0.048	8.59 M	4.27 G
WarpNet^*^ [11]	2022	0.157	0.838	0.038		
BinauralGrad [11]	2022	**0.128**	0.837	**0.030**	13.82 M	15.32 G
NFS [26]	2023	0.172	0.999	0.035	**0.55 M**	**357.58 M**
ZERO-SHOT [44]	2024	0.440	1.508	0.053	-	-
ours	2025	0.147	**0.789**	0.036	6.74 M	626.14 M

The results of WarpNet^*^ are given in [11]. The highest score is highlighted in bold and the second highest score is underlined.

**Table 4:** Comparison of perceptual evaluation metrics between our method and other binaural audio synthesis models.

Model	Year	MOS ↑	Spatialization ↑	Similarity ↑
GroundTruth	-	4.47 ± 0.147	4.31 ± 0.172	-
		-	-	-
DSP	-	3.48 ± 0.388	3.75 ± 0.192	3.64 ± 0.142
		3.23 ± 0.231	3.56 ± 0.167	-
WaveNet	2016	4.03 ± 0.263	3.78 ± 0.293	4.14 ± 0.264
		3.94 ± 0.25	3.67 ± 0.21	-
WarpNet	2021	4.01 ± 0.285	3.84 ± 0.228	4.04 ± 0.198
		4.00 ± 0.266 ^†^	4.05 ± 0.233	-
BinauralGrad	2022	4.04 ± 0.298 ^†^	4.13 ± 0.292 ^†^	4.19 ± 0.174 ^†^
		3.22 ± 0.189	3.7 ± 0.194	-
NFS	2023	3.13 ± 0.434	3.56 ± 0.23	3.93 ± 0.348
		3.99 ± 0.315	**4.27 ± 0.255**	-
ours	2024	**4.25 ± 0.287**	**4.17 ± 0.223**	**4.26 ± 0.169**
		**4.15 ± 0.219**	4.15 ± 0.215 ^†^	-

The highest score is highlighted in bold, while the second-highest score is indicated by †. For all models, the first row represents the test results of the binaural speech dataset, and the second row represents the test results of the out-of-domain audio.

**Table 5:** Ablation study results of proposed model.

Model	Wave-$l_{2}$↓	Phase-$l_{2}$↓	Amplitude-$l_{2}$↓
Ours	**0.147**	**0.789**	**0.036**
only TW	0.415	1.047	0.060
w/o POSA	0.199	0.846	0.043
w/o GCFM	0.157	0.813	0.040

## Data Availability

The original data presented in the study are openly available in https://openreview.net/forum?id=uAX8q61EVRu (accessed on 9 March 2025).

## Abbreviations

The following abbreviations are used in this manuscript:

VR	Virtual reality
AR	Augmented reality
ITD	Interaural time difference
ILD	Interaural level difference
DSP	Digital signal processing
HRIRs	Head-related impulse responses
BRIRs	Binaural room impulse responses
HRTFs	Head-related transfer functions
CP	Common portion
DP	Differential portion
POSA	POS-ORI self-attention module
GCFM	Gated-Conv fusion module
DS	Downsampling operation
US	Upsampling operation
SA	Self-attention mechanism
CA	Cross-attention mechanism
Pos	Position
Ori	Orientation
MSE	Mean squared error
STFT	Short-time Fourier transform operation
